# Modular Universal Tumor and Revision System Prostheses in Patients with Bone Cancer of the Lower Limbs: A Narrative Review of Functional Outcomes

**DOI:** 10.3390/cancers16193357

**Published:** 2024-09-30

**Authors:** Paola E. Ferrara, Mariantonietta Ariani, Sefora Codazza, Adelaide Aprovitola, Daniele Polisano, Gianpaolo Ronconi

**Affiliations:** 1 Department of Aging, Orthopedic and Rheumatological Sciences, University Polyclinic Foundation A. Gemelli IRCCS, 00100 Rome, Italy; paolaemilia.ferrara@policlinicogemelli.it (P.E.F.); sefora.codazza@guest.policlinicogemelli.it (S.C.); 2 Department of Neurosciences, Sense Organs and Thorax, Catholic University of the Sacred Heart, 00100 Rome, Italy; ade.apro@gmail.com; 3 Physical and Rehabilitation Medicine, University of Rome Tor Vergata, 00100 Rome, Italy; dr.dpolisano@gmail.com; 4 Department of Aging, Orthopedic and Rheumatological Sciences, Catholic University of the Sacred Heart, 00100 Rome, Italy; gianpaolo.ronconi@policlinicogemelli.it

**Keywords:** rehabilitations, MUTARS, megaprothesis, bone oncology

## Abstract

**Simple Summary:**

Primary bone tumors are rare, representing less than 0.2% of all malignancies. However, they constitute a relevant cause of disability due to their impact on the physical function and quality of life of affected patients. Surgical limb salvage is the first approach to bone tumors, whenever possible. The rehabilitative goals are reducing physical impairment and gaining functional independence. These goals must be equally considered to enable patients to become as independent as possible in their daily activities, achieving a sense of normalcy and well-being. This study aims to enhance the clinical practice approaches for patients with bone tumors treated with megaprostheses. Given this objective, we focused on three main outcomes: functional, surgical, and oncological.

**Abstract:**

The optimal management of bone tumors requires a multidisciplinary strategy to guarantee high-quality care. At specialized centers, the medical team responsible for managing patients with bone cancer comprises oncologists, surgeons, radiologists, pathologists, and rehabilitation specialists. The goal of treatment is to achieve long-term survival with minimal disability and pain. Postoperative rehabilitation is a fundamental therapeutic approach to enhance functionality and sustain the utmost quality of life following a limb-sparing surgery. Currently, megaprostheses are used for reconstructing bone defects after tumor resection, but in the literature, only a few studies have investigated rehabilitation outcomes in terms of functionality and impact on daily activities. This narrative review explores the functional and quality of life outcomes after the implantation of MUTARS^®^ prostheses in patients with lower extremity bone tumors. A comprehensive search was conducted on PubMed and Scopus using the following MESH terms: “MUTARS”, “Megaprosthesis”, “bone”, “tumors”, “metastasis”, “lower limb”, “rehabilitation”, “outcome”, and “quality of life”, and 10 studies were included. The most frequent oncological pathology was found to be primitive bone tumors treated with modular prostheses. The outcome measures used were the Henderson et al. classification, Harris Hip Scale, Musculoskeletal Tumor Society score, Visual Analog Scale, Range Of Motion, Karnofsky Performance Scale, and quality of life questionnaire. MUTARS^®^ is a well-established treatment option after bone tumor resection, although it involves extensive and complex post-resection reconstruction that exposes joints and tissues to substantial mechanical stress. Proper rehabilitation after MUTARS^®^ surgery is a fundamental therapeutic step, although there is still insufficient evidence in the literature focusing on functional and rehabilitative outcomes. Therefore, more studies and guidelines are needed to define standardized rehabilitation protocols for clinical practice after orthopedic oncologic surgery.

## 1. Introduction

Primary bone tumors account for less than 0.2% of all malignancies [1], while bone metastases, especially in adults, are much more common. The increasing knowledge of molecular profiling [2,3] and advances in diagnosis, therapies, and surgical techniques, including prosthesis design for reconstructing musculoskeletal defects, have opened up wider therapeutic opportunities, resulting in improved survival.

While therapies and techniques have improved, there has been minimal research on long-term functional outcomes and the well-being of patients. Research in this field is increasingly important to ensure patients’ quality of life and survival.

The three most common primary bone cancers are Osteosarcoma, Ewing sarcoma, and Chondrosarcoma, with different age and site distributions. Osteosarcoma occurs more often in children and young adults, with a higher occurrence at ages 10 to 14, and occurs most commonly around the knee. Ewing sarcoma is more common in teenagers and occurs most commonly in long bones. Chondrosarcoma typically occurs in patients 40 years and older and usually arises in the pelvis or long bones [4]. Among malignant bone tumors of the lower limbs, the distal femur and proximal tibia are the most often affected sites [5].

When possible, in patients with primary or metastatic bone tumors, a limb-sparing surgery is performed to replace the bone defect. The Modular Universal Tumor And Revision System (MUTARS^®^) is currently among the most used tumor and revision systems. Megaprostheses are modular prosthetic systems characterized by the peculiar adaptability of their constituent elements that can allow skeletal reconstructions following large and complex resections after bone tumors. It is thus possible to achieve anatomical reconstructions with lengths and angles that depend on the shape and extent of the bone lesions, thus allowing intraoperative adaptation to the individual patient’s situation [6,7]. It is worth noting that many complications occur after prosthesis replacement, as tumor surgery is a very traumatic surgery, including greater bone and soft tissue resection, blood loss, long operation times with a larger exposure range, which, combined with frequent radio- and chemotherapy, results in a higher rate of postoperative infections, and a higher risk of loosening and peri-prosthetic fractures. These complications also affect the patient’s rehabilitation and have a potential risk of secondary revision or amputation.

It is well known that therapeutic exercise and physical activity positively influence function in bone cancer patients [8,9], reducing post-surgical disability, improving independence in daily activities, and empowering the effectiveness of pharmacological cancer treatment.

This narrative review aims to describe the short- and long-term functional and rehabilitative outcomes of patients affected by primary or metastatic bone tumors in the lower limbs surgically treated with MUTARS^®^ prostheses to achieve a better understanding of the entire oncologic postoperative treatment pathway.

## 2. Methods

A comprehensive search was conducted on PubMed and Scopus using the MESH terms “MUTARS”, “Megaprosthesis”, “bone”, “tumors”, “metastasis”, “lower limb”, “rehabilitation”, “outcome”, and “quality of life”, in the context of human oncological disease, without additional restrictions.

A total of 24 articles were assessed for eligibility. After removing irrelevant studies—not concerning oncological patients or that have not cited the outcome measures of interest—we selected and analyzed 10 papers based on their titles and abstracts.

**Figure 1:**
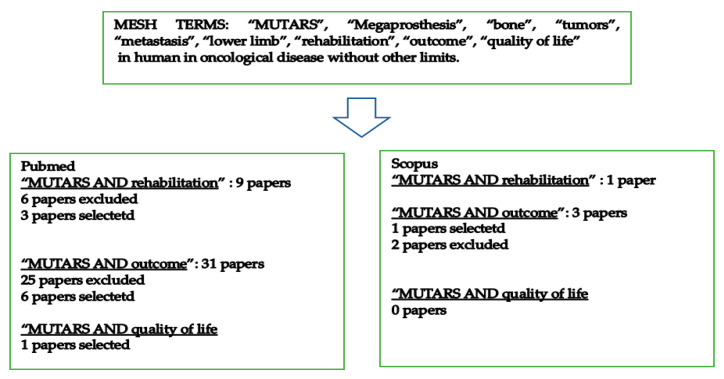
Search flowchart.

The data extracted from the selected studies were as follows: type of study (retrospective or prospective), patient characteristics (number, sex, and mean age), type of bone tumors of the lower limbs (histological characteristics and distribution between primary and metastatic lesions), the prosthesis model used for surgical replacement, the outcome measures, and the timing of the follow-up and the results. The main results are summarized in Table 1. The characteristics of this research are described in Figure 1.

## 3. Results

The analysis of the selected papers detailing functional outcomes of patients affected by lower limb primary and/or metastatic bone tumors treated with MUTARS^®^ is summarized in Table 1.

Our study comprised nine complete articles and one paper [19] evaluated based on title and abstract. We selected seven retrospective studies [10,11,13,14,15,19] and three prospective ones [12,17,18].

The retrospective analysis of the studies selected covers the period between 1992 and 2019. A total of 911 patients are included, including everyone surgically treated with modular prostheses of lower limbs. The sample size of the studies appears variable, ranging from a minimum of 21 [18] to a maximum of 250 subjects [13].

The sample’s demographic characteristics range from 4 years to 89 years, so this study covers both pediatric and adult age; the mean age of the study population is 42.1 years old, excluding one study [4] where no data are available. Contrary to major epidemiological data, females are more represented than males in the considered studies. The types of bone tumors included in the studies are disparate in histological terms and whether they are primary or secondary in nature. Two studies [11,13] analyze only primary tumors, and another two only study metastatic lesions [16,19]. In contrast, most studies collect data regarding primary and secondary bone lesions [10,12,18], while the remainder do not provide this information [14,15,17]. Histological tumors include metastatic bone tumors, Osteosarcoma, Leiomyosarcoma, Chondrosarcoma, giant cell tumors, and lymphomas. The most frequent tumor found is Osteosarcoma, according to current epidemiology.

Regarding the site of the MUTARS^®^ implant, most authors report prosthesis replacements of the knee [11,14,15,17] and hip region [12,16,19], while the remaining studies consider multiple sites of the lower limbs affected by bone tumors [10,13,18]. The available evidence in the literature shows which distal femur and proximal tibia are the most common sites of bone tumors in lower limbs [5]. The study presents a variety of modular endoprostheses, specifically 273 cemented, 45 uncemented, 154 silver coated, and 42 hydroxyapatite (HA) coated; however, these are not described in detail. Furthermore, some studies [16,17,19] also report using other megaprostheses implants (Global Modular Replacement System^®^; the Megasystem C^®^) without comparing them regarding the functional outcome.

The outcome measures used are heterogeneous from multiple points of view, showing considerable variability. Three papers [10,11,14] used the Henderson et al. classification to assess surgical outcomes, while four papers [10,11,14,15] used the classifications No Evidence of Disease (NED), Died With Disease (DWD), and Alive With Disease (DWD) to analyze oncological outcomes. The rehabilitation outcomes of this study are summarized as follows: as functional measures, three studies used the Harris Hip Scale (HHS) [12,16,19], one study used the Modified Harris Hip Scale Modified (m-HHS) [12], and one study used the Oxford Knee Score (OKS) [20]. As objective measures, two studies employed gait analysis [9,11], and one study used stabilometry and Short Physical Performance Battery (SPPB) [18]. Four studies used the Visual Analog Scale (VAS) for subjective pain [12,16,18,19]; four studies used the Range Of Motion (ROM) of the considered joint [14,15,18]; and one study used the SF-36 questionnaire to assess quality of life [17]. Some studies use functional measures specifically for a particular patient population: Musculoskeletal Tumor Society score (MSTS) was reported in seven studies [10,13,14,16,17,18,19], the Karnofsky Performance Scale (KPS) was used in two studies [15,18], the Enneking score was calculated in one study [13], and both the Toronto Extremity Salvage Score (TESS) and Eastern Cooperative Oncology Group (ECOG) were included in the same study [18].

## 4. Discussion

Most of the studies investigated the survival of the implants, the incidence, and the types of post-surgical complications after modular replacement to reduce complications and maximize the effectiveness of surgical treatment. The rehabilitation goals and protocols in the pre-operative and postoperative phases have not been sufficiently investigated. Although a homogeneous comparison of all studies is unfeasible because of the heterogeneity of the demographic data and the follow-up time of the sample, we contextualized rehabilitation since function cannot be separated from the assessment of surgical and oncologic outcomes.

### 4.1. Functional Outcomes

The most cited functional measure is MSTS [10,16,18,19,20], a well-accepted psychometric properties scoring system [20]. The studies report excellent MSTS results in those with a lower limb megaprosthesis implanted. In the study by Lopresti et al., MSTS showed statistically significantly higher scores in patients who underwent a physiotherapy program after surgery. In Hardes J et al.’s study [14], they considered not only the MSTS but also the OKS. They observed that although the extra-articular resection achieved good function, the rates of complications and subsequent amputation were higher than in patients treated with intra articular resection. They also reported that despite the good functional results, several patients needed support when walking and had problems kneeling, limping, and descending stairs.

According to MSTS (mean value was 79.2 ± 3.9%), the study by Pellegrino et al. showed that a significant percentage of patients reached a medium–high functional outcome and satisfaction after rehabilitation. Notably, the category that obtained superior results was ‘stability’, while poor results heavily influenced the scores in the ‘strength’ and ‘emotional acceptance’ categories. Information regarding the quality of life is reported in the study by Hardes et al. The employability of their sample after the intervention was reported as follows: out of the 21 patients evaluated, 52% (*n* = 11) worked full-time, and only one could not work at all.

The reduction in pain was evaluated with the Visual Analogical Scale in four studies [2,4,7,11]. The results demonstrated low pain levels after the implant and, at the same time, good physical performance. Guzik et al. demonstrated an accordance between function improvement (evaluated by KPS and MSTS) and pain.

Three studies [12,16,19] used the HHS to assess functional outcomes. Ref. [12] also evaluated functional outcomes using the modified-HHS. The HHS is a reliable assessment that addresses pain, function, absence of deformity, and physical examination associated with the range of motions.

The study by Kamiński et al. compares the outcomes of two groups of patients who received MUTARS femoral resection prostheses: one group was diagnosed with tumor metastasis, and the other underwent revision surgery due to implant loosening.

The first observation is that the second group needed longer hospitalization after surgery. This may be attributed to the impact of multiple surgical procedures on the musculoskeletal system, which resulted in motor deconditioning and the need for longer rehabilitation. The study describes a higher incidence of complications in the second group, namely dislocations (8 vs. 2) and cases of surgical site infection. Both groups showed, however, similar improvements in pain and HHS score at follow-up (one year after the procedure). Both groups recorded improvements in physical ability, except for prolonged comfortable sitting, putting on shoes and socks, and the capacity to use public transportation, all of which affect participation in daily activities. Also, patients who underwent arthroplasty revision required more focus on pain reduction during everyday tasks such as walking and climbing stairs.

With regard to gait alterations after surgery, in one study [16], 15 out of 64 patients ambulated efficiently without crutches, 39 patients used one crutch or a walking cane when walking longer distances, and 10 patients walked with two crutches. Muscle strength of the operated limb was lower in all patients. An improved gluteus function was indicated by observing a positive Trendelenburg’s sign. The patients could use stairs with alternating gait (37 patients) or lead with the healthy limb and follow with the affected limb (27 patients); there were no knee contractures, making it difficult or impossible for them to rise from a chair. Gait analysis is investigated in the study by Pellegrino et al. They compared gait alterations between patients who underwent megaprosthetic replacement for tumors around the knee and patients who underwent total knee arthroplasty (TKA) for osteoarthritis (OA). Standard TKA implants and MUTARS result in similar gait alterations, characterized by reduced walking speed compared to healthy people. However, gait abnormalities do not significantly affect megaprosthetic replacement than TKA implants. The compensatory mechanisms for gait speed differ between the two groups: in the cancer group, the disparity in stance is counterbalanced by the healthy limb; in the OA group, the limbs tend to adjust via adopting similar stance values, resulting in a reduction in speed and an increase in the double stance phase. One study [8] reported that at a mean of 13.2 years from the endoprosthetic reconstruction, patients with proximal/distal femoral replacements and proximal tibia replacements all walked efficiently. Patients with proximal tibia replacements had more muscle weakness around the knee, but all groups remained similarly active at home and in the community.

ROM is evaluated in four studies [14,15,17,18]. Pellegrino et al. investigated ROM of the knee in patients with megaprosthetic replacements due to tumors, comparing them with patients who underwent total knee arthroplasty due to osteoarthritis, with no significant difference between the two implants. Ferrara et al. investigated the hip and knee flexion range of movement in patients with MUTARS^®^ reconstructions after proximal or distal lower limb tumor resection. The results showed a significant improvement in hip flexion ROM at the second follow-up three months after rehabilitation. A near-significant increase in quadriceps muscle strength was observed between T1 and T2, with a *p*-value of 0.08. The improvement of ROM and muscle strength was progressive, as were the self-sufficiency and psychophysical conditions evaluated using SPPB, MSTS, and TESS in the first six months after surgery. Although the ROM after the MUTARS^®^ implant was somewhat restricted due to surgical factors and the structural requirements of the prosthesis, researchers observed a substantial improvement in hip flexion ROM three months after surgery. In one study [14], after intra articular knee resection, an active extension deficit of more than 10° was reported in only 6 out of 98 patients (6%). In another study [15], after extra-articular knee resection, an extensor lag > 10° was noted in 10 out of 59 patients (17%), resulting in a functional deficiency.

At stabilometry evaluation, Ferrara et al. reported a significant balance increase at the follow-up two and three months after surgery. The SPPB values increased (*p*-value 0.09) at three months and increased more significantly (*p*-value 0.01) at six months after surgery, with better results in balance, walking time, and “sit to stand” ability. Moreover, the study showed a significant increase in the TESS scale (*p*-value 0.03) three months after surgery.

Day-to-day activities are described only in two studies, and the results obtained using the KPS [15,18] and ECOGG [18] scales were all positive. These studies provide an understanding of the fundamental activities of daily living required for independent care, which has possibly the highest impact on quality of life. It is important to note that quality of life is only investigated in one study [17]. Pellegrino et al. demonstrated that modular prostheses considerably improved patients’ quality of life. Employability after intervention was also investigated [15]. Harders J et al. demonstrated that 52% of their study population worked full-time after recovery, and only one could not work.

### 4.2. Surgical Outcomes

Surgical complications are reported in three studies [10,11,14], while one study [15] classified them as major (peri-prosthetic infection, aseptic loosening, peri-prosthetic fracture) or minor (change in the bushings, wound healing disturbances).

Reducing the rate of infections has been widely studied. As detailed in the literature [21,22,23], materials such as iodine, Defensive Antibacterial Coating (DAC), or Silver Coating reduce the risk of infection, avoiding biofilm formation on the implant surface. Pala et al.’s study supported this evidence, showing that silver-coated MUTARS^®^ prostheses are related to a lower incidence of infection, even if not statistically significant, as they inhibit bacterial colonization of the prosthetic body without toxicological side effects. Therefore, silver-coated prostheses are preferable in higher-risk patients, particularly in sites such as the distal femur and proximal tibia, where they prevent infections more effectively than in other sites. They reported that infections were more frequent in the lower limbs than in the upper limbs, with no difference in survival due to infection (*p*-value 0.76). They also reported that silver-coated prostheses, two-stage revision, and prostheses implantation showed a lower secondary amputation incidence than titanium implants.

Another consideration comes from Hardes et al.’s study [15]. They found out that extra-articular knee resection, as an alternative to amputation, had a higher rate of delayed wound healing and associated peri-prosthetic infections than intra articular resection. Because of peri-prosthetic infection and revision, one patient had a flexion limitation of 20° in the ROM of the knee. In 38 patients with no infection at final follow-up, the mean range of flexion was 72° (10° to 100°), and a total of 25 had flexion of ≥90°. In two cases, the infection and necrosis were extensive to the point that it was necessary for amputation. In this regard, patients who developed peri-prosthetic infections were the ones who received radiotherapy, resulting in delayed wound healing. This also agrees with Hardes et al.’s study [15], which reported that a high BMI and administration of radiotherapy are significant risk factors for developing wound healing disturbances.

In the literature, patellar tendon rupture (Type 1) is reported as a common complication in knee replacements [24]. Patellar tendon ruptures were not observed in Bus et al.’s study, likely due to the use of the attachment tube, which enables the extensor apparatus to ingrow and guarantees a dependable, long-lasting fixation.

They also reported that uncemented HA-coated distal femoral replacements had a lower risk of loosening complication (Type 2) (5%) than uncemented uncoated implants (31%) (*p*-value 0.060).

In two groups of patients with femoral resection prostheses (tumor metastasis and implant loosening) in Kamiński et al.’s study, MUTARS^®^ stems, both press-fit cementless and cemented, allowed immediate weight bearing. This is particularly important; not delaying the limb load soon after the procedure improves the patient’s quality of life and facilitates the continuation of specialist treatment of the underlying disease.

Another consideration comes from Gosheger et al.’s study, which reported that the stem’s hexagonal-shaped design provides good rotational stability, reduced loosening rates, and less stem breakage. Also, using the Trevira tube (Implantcast) makes muscle and tendon refixation much easier, decreases dislocation, and improves functional results in proximal femoral and tibia replacements.

Three studies also evaluated the limb survival rate [12,14,15] using the Kaplan–Meier survivorship analysis. In one of these [13], the 5-year limb survival rate evaluated was 87.1%. In the second [14], the limb survival rates were 94.9%, 90.5%, and 74.5% at one, two, and ten years, respectively. In the third [15], the limb survival rate was 76% at 151 months, while the prosthesis survival rate (without re-operation) was 48% at two years and 25% at five years post-operatively

In the first of these studies, the authors demonstrated that radiation therapy and chemotherapy harmed prosthetic survival. The second study specifically identified radiotherapy (*p* = 0.041), pathological fracture (*p* = 0.004), peri-prosthetic infection (*p* = 0.018), and the use of a reattachment tube (*p* = 0.01) as independent significant risk factors for subsequent amputation on univariate analysis

### 4.3. Oncological Outcomes

Only four studies [10,11,14,15] described the oncologic outcomes at follow-up. The timing of follow-up is different between the four studies: mean follow-up is 3.4 years [10], 1 year [14], and 4.7 years while median follow-up is 8.9 years [11].

We noted that, regardless of the timing of follow-up, in Pala et al.’s study, the percentage of patients with NED is well below the other two studies [14,15]. Likewise, the DWD and the AWD outcomes were different.

Regarding overall survival at 5 and 10 years, we also noticed a lower percentage of patients in Pala et al.’s study (58% at 5 years; 54% at 10 years) compared to Hardes et al.’s study (84.9% at 5 years; 80.1% at 10 years). We attributed some of this evidence to the mean age of the samples in the respective studies (53 vs. 18 years old).

## 5. Conclusions

In conclusion, MUTARS^®^ implantation is a good alternative for patients with primary or metastatic bone tumors. Extensive resections and prosthetic reconstructions inevitably change the alignment, biomechanics, and proprioception of the affected limb, resulting, for example, in changes in walking patterns. MSTS is a validated functional measure for orthopedics and oncologists, but more functional outcome assessments are needed to identify specific problems patients face. This information can be used to create rehabilitation protocols to optimize patient care after limb reconstruction and achieve not only a lower rate of complications but also a satisfactory quality of life after cancer.

## Figures and Tables

**Table 1:** Description of the included studies.

Authors	Type of Study	Patients	Bone Tumor Lower Extremity	Model of Prosthesis	Outcome Measures	Timing	Results
Pala et al. (2021) [10].	Retrospective; multicentric.	*n*: 187; M/F: 100/87; Mean age (range): 53 years (4–89).	*n*: 107 Malignant bone tumors or soft tissue tumors with bone involvement; *n*: 52 Metastatic; *n*. 13 Lymphomas/Myelomas; *n*. 8 Giant cell tumors; *n*. 7 Non-oncologic.	MUTARS; Upper/Lower limbs: 72/115; Regarding Lower limbs: *n*: 56 Cemented; *n*: 59 Non-cemented; *n*: 65 Silver-coated.	Oncological outcomes: *n*: 143 Surgical complications (Henderson et al.); *n*: 139 MSTS.	2000–2019; Mean oncologic follow-up: 3.4 years (range: 1 month–16 years).	MSTS after surgery: Average score: 25.1 (9–30); *n*: 115 (excellent); *n*: 20 (good); *n*: 4 (fair); *n*: 0 (poor).
Bus et al. (2015) [11].	Retrospective.	*n*: 101; M/F: 55/46; Mean age (range): 36 years (13–82).	*n*: 56 Osteosarcoma; *n*: 10 Leiomyosarcoma; *n*: 9 Chondrosarcoma; *n*: 9 Giant cell tumors; *n*: 7 Pleomorphic undifferentiated sarcoma; *n*: 5 Ewing sarcoma; *n*: 2 Low-grade Osteosarcoma; *n*: 2 Sarcoma not otherwise specified; *n*: 1 Synovial sarcoma; *n*: 1 Diffuse-type giant cell tumor.	MUTARS; Knee; Cemented/Non-cemented: 23/78; Hydroxyapatite-coated: 42.	Oncological outcomes: *n*: 63 Surgical complications (Henderson et al.).	1995–2010; Median follow-up: 8.9 years (range: 8.0–9.7).	Absence of rehabilitation outcomes.
Kamiński et al. (2017) [12].	Prospective cohort study.	*n*: 34; M/F: 12/44; Mean age (range): 72 years (45–85).	*n*: 13 Metastasis; *n*: 21 cases: Non-oncological prosthesis revision.	MUTARS; Hip; Cemented: 34.	HHS; M-HHS; VAS.	Dec. 2008 to Jan 2016; Follow up on the first postoperative day and at 3, 6, and 12 months.	There is an absence of statistically significant differences between groups in HHS, m-HHS, and VAS after surgery, although similar improvements in both groups.
Gosheger et al. (2006) [13].	Retrospective.	*n*: 250; M/F: 135/115; Mean age (range): 30.7 years (7.4–80).	*n*: 139 Osteosarcoma; *n*: 43 Chondrosarcoma; n: 36 Ewing sarcoma; *n*: 15 Pleomorphic sarcoma; *n*: 6 Parosteal osteosarcoma; *n*: 3 Leiomyosarcoma; *n*: 4 Soft tissue sarcoma with bone involvement; *n*: 4 Giant cell tumor.	MUTARS; Upper/Lower limbs: 51/199; Regarding Lower limbs: *n*: 58 Cemented; *n*: 141 Non-cemented; Since 1997, all HA-coated; All cemented prostheses contain gentamicin.	MSTS.	1992–2003; Mean follow-up: 45 months (range: 3–140 months).	Average MSTS (lower limb): 25, after proximal tibia replacement (range 13–30); 24, after distal femoral replacement (range 8–30); 21, after proximal femoral (range 14–29); 20, after total femoral replacements (range 13–27). Average MSTS (upper limb): 23, after distal humerus replacement (range 18–27); 21, after humerus replacements (range 1–25); 19 after total humerus replacements (range 18–20).
Hardes et al. (2018) [14].	Retrospective.	*n*: 98; Median age (range): 18 years (10–78).	*n*: 63 Osteosarcoma; *n*: 16 Ewing sarcoma; *n*: 6 Pleomorphic sarcoma; *n*: 6 Giant cell tumor; *n*: 5 Chondrosarcoma; *n*: 1 Leiomyosarcoma; *n*: 1 Parosteal osteosarcoma.	MUTARS; Knee; *n*: 9 Tibia component (cemented) and hybrid-fixated femur component (cementless stem with cemented shield); Silver-coated: 56.	Oncological outcomes: Surgical complications (Henderson et al.); Knee extension in patients after surgery: *n*: 51 No deficit; *n*: 11 5°–10° Deficit; *n*: 6 > 10° Deficit. Knee flexion in patients after surgery: *n*: 55 ≥ 90°; *n*: 9 89°–80°; *n*: 4 40°–70°.	1996–2014; Mean oncological follow-up: 45 months (range: 3–140 months).	Absence of rehabilitation outcomes; There were no statistically significant associations between an active extension deficit and patella alta. In contrast, patella baja was associated with a noticeable reduction in patients with flexion > 90°.
Hardes et al. (2013) [15].	Retrospective.	*n*: 59; M/F: 36/23; Mean age (range): 33 years(11–74).	*n*: 34 Osteosarcoma; *n*: 7 Chondrosarcoma; *n*: 7 Synovial sarcoma; *n*: 7 Pleomorphic sarcoma; *n*: 3 Leiomyosarcoma; *n*: 1 Giant cell tumor of the patella.	MUTARS; Knee (distal femur, proximal tibia); *n*: 14 Femur component (cemented); Silver-coated: 33.	Oncological outcomes: Surgical complications (classified as major and minor); *n*: 46 MSTS; *n*: 21 OKS; *n*: 38 ROM of the knee.	1992–2011; Mean follow-up: 62 months (12 to 211).	Mean MSTS (range): 22 (10 to 29); Mean OKS (range): 32 (10 to 48); Mean range of flexion (range): 72° (10° to 100°). A total of 25 of these had flexion of ≥ 90°, and only one had gross limitation of movement with flexion of 20° after peri-prosthetic infection and revision. An orthosis and/or a walking aid were used by 12 patients.
Guzik (2016) [16].	Retrospective.	*n*: 64; M/F: 38/26; Mean age in F: 66 years; Mean age in M: 69 years; In total, 64% of patients had pathological fractures and were unable to walk.	*n*: 64 Metastatic lytic tumors.	*n*: 36 MUTARS; Hip (proximal femur); *n*: 28 GMRS; Cemented/Non-cemented: 19/45.	MSTS; VAS; HHS; KPS.	2010–2014; Mean follow-up (range): 1.8 (3.6 to 1.2) years.	Six weeks after surgery: Mean MSTS: 20 (18–21); Mean VAS: 3.8 (2–5); Mean HHS: 75 (71–81); Mean KPS: 64 (50–80). Twelve weeks after surgery: Mean MSTS: 21 (18–22); Mean VAS: 3.4 (2–5); Mean HHS: 81 (71–86); Mean KPS: 65 (50–80). Walking: *n*: 15 patients could ambulate efficiently without crutches; *n*: 39 patients could use one crutch or a walking cane when walking over longer distances; *n*: 10 patients could walk with two crutches; Muscle strength of the operated limb: Lower in all patients; Use of stairs: *n*: 37 patients had an alternating gait; *n*: 27 patients led with the healthy limb and followed with the affected limb.
Pellegrino et al. (2020) [17].	Observational case–control study.	*n*: 26 in the oncological group (case); M/F: 13/13; Mean age ± SD (range): 40.9 ± 18.9 (range: 15–75). VS *n*: 21 in the osteoarthritis group (control); M/F: 8/13; Mean age ± SD (range): 68.0 ± 4.7 (range: 56–74).	*n*: 12 Osteosarcoma; *n*: 5 Chondrosarcoma; *n*: 4 Giant cell tumor; *n*: 2 Undifferentiated sarcomas; *n*: 2 Leiomyosarcomas; *n*: 1 Primitive bone lymphoma.	Oncological group: *n*: 10 GMRS; *n*: 9 LINK^®^; *n*: 7 MUTARS. VS Osteoarthritis group: TKA with a posterior stabilized, ultra-congruent, or cruciate retaining implant.	Gait analysis (basography, knee ROM, electromyographic activity of some group of muscles during the gait cycle); ROM of the knee; MSTS (only oncological group) SF-36.	Oncological group: 2006–2016 (minimum follow-up of 12 months); Osteoarthritis group: 2010–2014 (minimum follow-up of 12 months).	Gait analysis: Mean speed (m/s ± SD): Oncological/osteoarthritis: 0.83 ± 0.22/0.76 ± 0.21; Cadence (stride/min ± SD): Oncological/osteoarthritis: 47.8 ± 5.4/45.3 ± 6.6; No statistically significant differences were detected between surgical approaches in the oncological group; ROM of the knee: There was a statistically significant difference between the healthy and the operated limbs in both groups. However, no significant difference was registered between the limb with megaprosthesis and the limb with a standard implant. Mean MSTS (% ± SD): 79.2 ± 3.9 SF-36 (subscale): The mean value was higher in the oncological group in terms of bodily pain, vitality, social functioning, and mental health; The mean value was higher in the osteoarthritis group regarding general health.
Ferrara et al. (2019) [18].	Observational.	*n*: 21; M/F: 7/14; Mean age ± SD: 61.76 ± 14.68.	*n*: 15 Metastatic bone tumor; *n*: 6 Osteosarcoma.	MUTARS; 71.4% proximal femur; 23.8% distal femur; 4.8% both.	ROM; VAS; SPPB; ECOG; KPS; MSTS; TESS; Stabilometry.	February 2017–December 2018; Follow-up at one week, one month, three months, six months, and one year.	Significant improvement: VAS at T1 hip ROM, MSTS, and TESS at T2; SPPB at T3; No significant results in stabilometry.

MUTARS: Modular Universal Tumor And Revision System; GMRS: Global Modular Replacement System; TKA: total knee arthroplasty; MSTS: Musculoskeletal Tumor Society score; HHS: Harris Hip Score; M-HHS: modified-HHS; VAS: Visual Analog Scale; OKS: Oxford Knee Score; KPS: Karnofsky Performance Scale; ECOG: Eastern Cooperative Oncology Group; TESS: Toronto Extremity Salvage Score; SPPB: Short Physical Performance Battery; SF-36: Short Form Health Survey-36.

## Data Availability

The data generated and analyzed during this study are included in this published study and are available from the corresponding author.
